# Identification of Rhoptry Trafficking Determinants and Evidence for a Novel Sorting Mechanism in the Malaria Parasite *Plasmodium falciparum*

**DOI:** 10.1371/journal.ppat.1000328

**Published:** 2009-03-06

**Authors:** Dave Richard, Lev M. Kats, Christine Langer, Casilda G. Black, Khosse Mitri, Justin A. Boddey, Alan F. Cowman, Ross L. Coppel

**Affiliations:** 1 The Walter and Eliza Hall Institute of Medical Research, Parkville, Victoria, Australia; 2 Department of Microbiology, Monash University, Victoria, Australia; University of Georgia, United States of America

## Abstract

The rhoptry of the malaria parasite *Plasmodium falciparum* is an unusual secretory organelle that is thought to be related to secretory lysosomes in higher eukaryotes. Rhoptries contain an extensive collection of proteins that participate in host cell invasion and in the formation of the parasitophorous vacuole, but little is known about sorting signals required for rhoptry protein targeting. Using green fluorescent protein chimeras and *in vitro* pull-down assays, we performed an analysis of the signals required for trafficking of the rhoptry protein RAP1. We provide evidence that RAP1 is escorted to the rhoptry via an interaction with the glycosylphosphatidyl inositol-anchored rhoptry protein RAMA. Once within the rhoptry, RAP1 contains distinct signals for localisation within a sub-compartment of the organelle and subsequent transfer to the parasitophorous vacuole after invasion. This is the first detailed description of rhoptry trafficking signals in *Plasmodium*.

## Author Summary

The malaria parasite *Plasmodium falciparum* is a eukaryotic organism with multiple membrane bound organelles with discrete functions. The rhoptry is an unusual secretory organelle that participates in host cell invasion and the formation of a specialised vacuole that the parasite occupies during the intracellular part of its lifecycle. Rhoptries contain an extensive collection of proteins, but little is known about how these proteins are trafficked to their destination. Understanding determinants of rhoptry protein trafficking will help us to identify novel rhoptry proteins, and may provide targets for therapeutic intervention. In the current study, we focussed on the trafficking of the rhoptry protein RAP1. By making parasites that express regions of RAP1 fused to Green Fluorescent Protein (GFP), we were able to map in detail the domains of RAP1 that are necessary for correct trafficking. We also provide evidence that RAP1 is targeted to rhoptries via its interaction with another rhoptry protein, RAMA. This is the first detailed description of rhoptry trafficking signals in *Plasmodium*.

## Introduction

Malaria is a disease that causes severe morbidity, mortality and socio-economic hardship in tropical and sub-tropical areas of Africa, South America and Asia. *Plasmodium falciparum* causes the most serious form of the disease and is responsible for more than 2 million deaths annually [1]–[3]. The development and implementation of novel intervention strategies in the form of drugs, vector control measures and an effective vaccine remains an urgent global health priority [4].

*Plasmodium* spp. belong to the phylum Apicomplexa – protozoan parasites characterised by a complex lifecycle consisting of invasion followed by rounds of intracellular replication. The invasion is mediated by a set of molecules distributed on the parasite surface and within specialised apical secretory organelles. Regulated secretion from these organelles allows the parasite to adhere to an appropriate target cell, invade and induce the formation of a specialised parasitophorous vacuole (PV) in which it subsequently resides (reviewed in [5]).

Rhoptries are the largest of the *Plasmodium* secretory organelles and contain more than 20 proteins, many of which are unusual and have no recognisable orthologues, even in the closely related apicomplexan parasite *Toxoplasma gondii* (reviewed in [6]). Rhoptries are pear-shaped and membrane bound, and in transmission electron micrographs the bulb and neck appear to form distinct sub-compartments. The neck is electron-lucent while the bulb is electron-dense and contains internal membranes reminiscent of multivesicular endosomes in higher eukaryotes [7]–[9]. Individual proteins are not distributed throughout the rhoptry but are sub-compartmentalised within either the bulb or the neck [10]–[12].

Rhoptry biogenesis occurs by sequential fusion of Golgi-derived vesicles which deliver protein cargo into the rhoptry lumen [9],[13]. Rhoptry proteins pass through the endoplasmic reticulum (ER) and the Golgi [14],[15], but specific targeting signals which direct protein sorting into rhoptry destined vesicles remain poorly understood. In mammalian cells, sorting of transmembrane proteins is mediated by cytoplasmic adaptor complexes (APs) which recognise specific motifs (e.g. the YXXΦ motif, where Φ is a hydrophobic amino acid) within their cytoplasmic tails. APs select cargo for inclusion into a transport vesicle and recruit coat components (e.g. clathrin) necessary for vesicular budding and transport [16],[17]. This mechanism has been shown to operate in *Toxoplasma*, and may also be conserved in *Plasmodium* [18],[19]. However, most *Plasmodium* rhoptry proteins described to date do not possess a transmembrane region and cytoplasmic tail, implying the existence of additional sorting pathways [6].

One possibility is that sorting within the Golgi occurs via a clustering mechanism whereby proteins en route to a particular destination aggregate into distinct sub-domains [20]. The rhoptry associated membrane antigen (RAMA) is a glycosylphosphatidyl inositol (GPI)-anchored protein that is expressed early in the asexual red blood cell (RBC) cycle. Most rhoptry proteins are expressed at the late trophozoite stage but RAMA is first synthesised during the late ring stage, before the appearance of recognizable rhoptries, and appears to temporarily accumulate within compartments of the secretory pathway [15]. This unusual expression pattern suggests that RAMA may be involved in rhoptry biogenesis and protein targeting. Fluorescence Resonance Energy Transfer (FRET) experiments indicate that RAMA interacts with the low molecular weight (LMW) rhoptry complex [15]. The LMW complex is a heterodimer composed of rhoptry associated protein 1 (RAP1), and RAP2 or RAP3 [21]. Rhoptry targeting of the LMW complex occurs via the N-terminus of RAP1, although the mechanism is not understood [22]. We hypothesised that RAMA acts as an escorter for RAP1 to recruit RAP1, −2 and −3 into a rhoptry-destined protein complex.

Here we have used expression of heterologous reporter constructs and pull-down assays to map the RAP1 targeting signals and define the RAMA-RAP1 interaction. Our results provide evidence of a novel mechanism for trafficking of proteins to this unusual secretory organelle.

## Results

### RAP1 contains a bipartite rhoptry targeting signal

In *P. falciparum* schizont stage parasites, RAP1 is localised in the rhoptry bulb [21],[23]. Previously, it has been shown that the first 344 amino acids of RAP1 are sufficient for rhoptry targeting [22]. To more precisely define these targeting signals, we used constructs consisting of regions of RAP1 fused to green fluorescent protein (GFP). GFP was chosen as a reporter because it has previously been used in a variety of studies in *Plasmodium* and does not possess any endogenous targeting signals. When expressed on its own, GFP localises to the parasite cytoplasm. However, addition of sorting signals can result in trafficking of GFP to compartments of the secretory system [24],[25]. The expression of RAP1-GFP chimeras was driven by an inducible promoter with a pattern of expression similar to merozoite surface protein 2 (MSP2) [26]. A late stage promoter was selected to avoid aberrant targeting as a result of incorrect timing of expression [27],[28].

**Figure 1:**
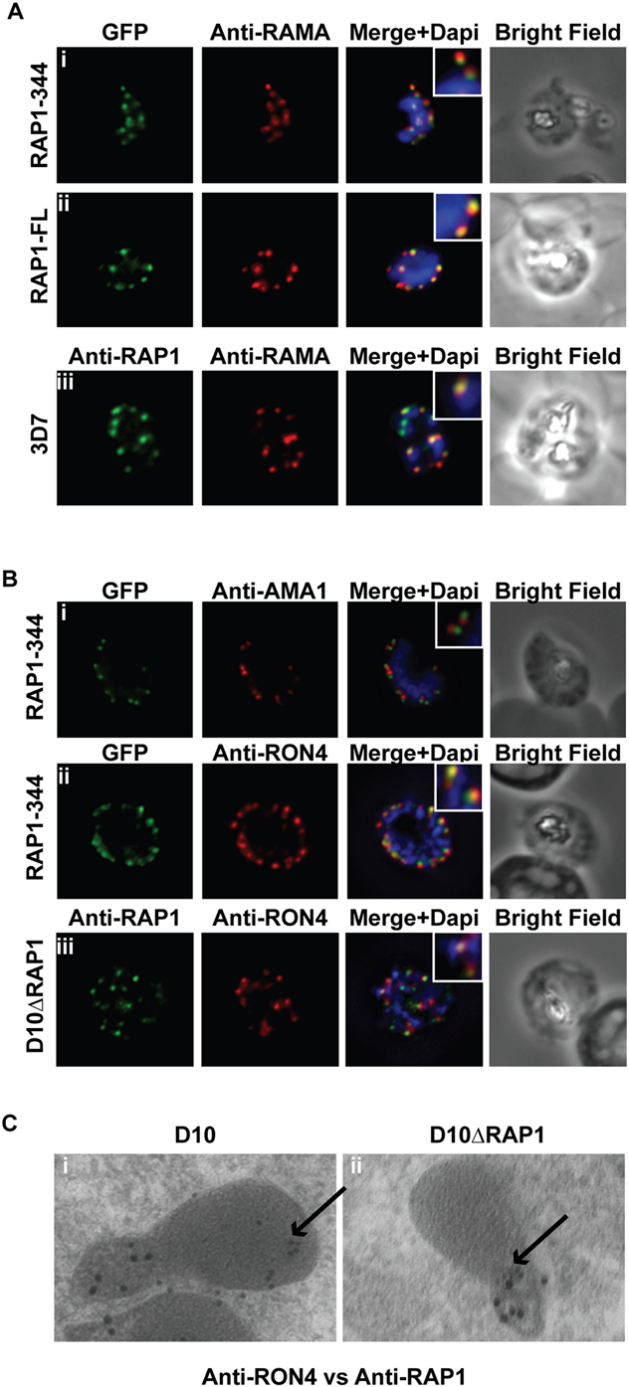
RAP1 contains a bipartite rhoptry signal. (A) RAP1-344 and RAP1-FL GFP fusions. Both constructs show a punctate fluorescence pattern characteristic of rhoptry localisation. For the RAP1-FL construct, GFP signal overlaps with RAMA. In contrast, the RAP1-344GFP chimera only partially overlaps with RAMA, suggesting rhoptry neck localisation. (B) For the RAP1-344 construct, GFP co-localises with the rhoptry neck marker PfRON4 and does not co-localise with the microneme marker AMA1. Likewise in the transgenic parasite line D10ΔRAP1, truncated RAP1 co-localises with PfRON4. (C) Immunoelectron microscopy demonstrates that truncated RAP1 (10 nm beads) in D10ΔRAP1 parasites is localised in the rhoptry neck, whereas full-length RAP1 in D10 (wild-type) parasites is localised in the rhoptry bulb. PfRON4 (15 nm beads) is localised in the rhoptry neck in both parasite lines.

To verify that GFP could be trafficked to rhoptries, we initially generated two constructs – GFP fused to amino acids 1-344 of RAP1 (RAP1-344) and GFP fused to the entire RAP1 sequence (RAP1-FL). The constructs were introduced into *P. falciparum* 3D7 parasites and the trafficking of GFP was followed using fluorescence microscopy (Figure 1A). As expected, both constructs produced a punctate pattern of staining in schizonts, characteristic of localisation within the apical secretory organelles. Surprisingly however, the two constructs demonstrated subtly different localisation patterns. For RAP1-FL, GFP fluorescence co-localised with the rhoptry bulb marker RAMA [15], and mimicked the localisation of native RAP1 in wild-type parasites (Figure 1Aii and 1Aiii). In contrast, for RAP1-344, GFP staining only partially overlapped and was anterior to a larger RAMA-positive structure (Figure 1Ai). To ascertain the precise localisation of the RAP1-344GFP chimera, we performed double labelling experiments with PfRON4 (a rhoptry neck marker) [29] and apical membrane antigen 1 (AMA1, a microneme marker) [30] using specific antibodies (Figure 1B). Our results strongly suggest that for RAP1-344, GFP is not localised in micronemes (Figure 1Bi) but is localised in the rhoptry neck (Figure 1Bii).

To explore whether rhoptry neck localisation of truncated RAP1 was an artefact of our heterologous expression system, we reanalysed the original *rap1* truncation mutant (D10ΔRAP1)[22]. This mutant, generated by single cross-over homologous recombination in the parasite line D10, has a truncated *rap1* gene expressing amino acids 1-344 of RAP1 under the control of its native promoter. Interestingly, the same pattern was observed for D10ΔRAP1 as for RAP1-344, with GFP co-localising with PfRON4 (Figure 1Biii). To further confirm this finding, we localised RAP1 in D10ΔRAP1 and its parent line using immunoelectron microscopy (Figure 1C). In D10, native RAP1 is localised in the rhoptry bulb, whereas in D10ΔRAP1 the truncated RAP1 protein is localised in the rhoptry neck, adjacent to PfRON4. Taken together, this data strongly suggests that RAP1 contains a bi-partite rhoptry signal: amino acids 1-344 are sufficient for targeting RAP1 to the rhoptry and amino acids 344-782 are necessary to avoid re-localisation of the protein from the bulb of the rhoptries to the neck.

### Amino acids 22-55 of RAP1 are sufficient for targeting GFP to the rhoptries

**Figure 2:**
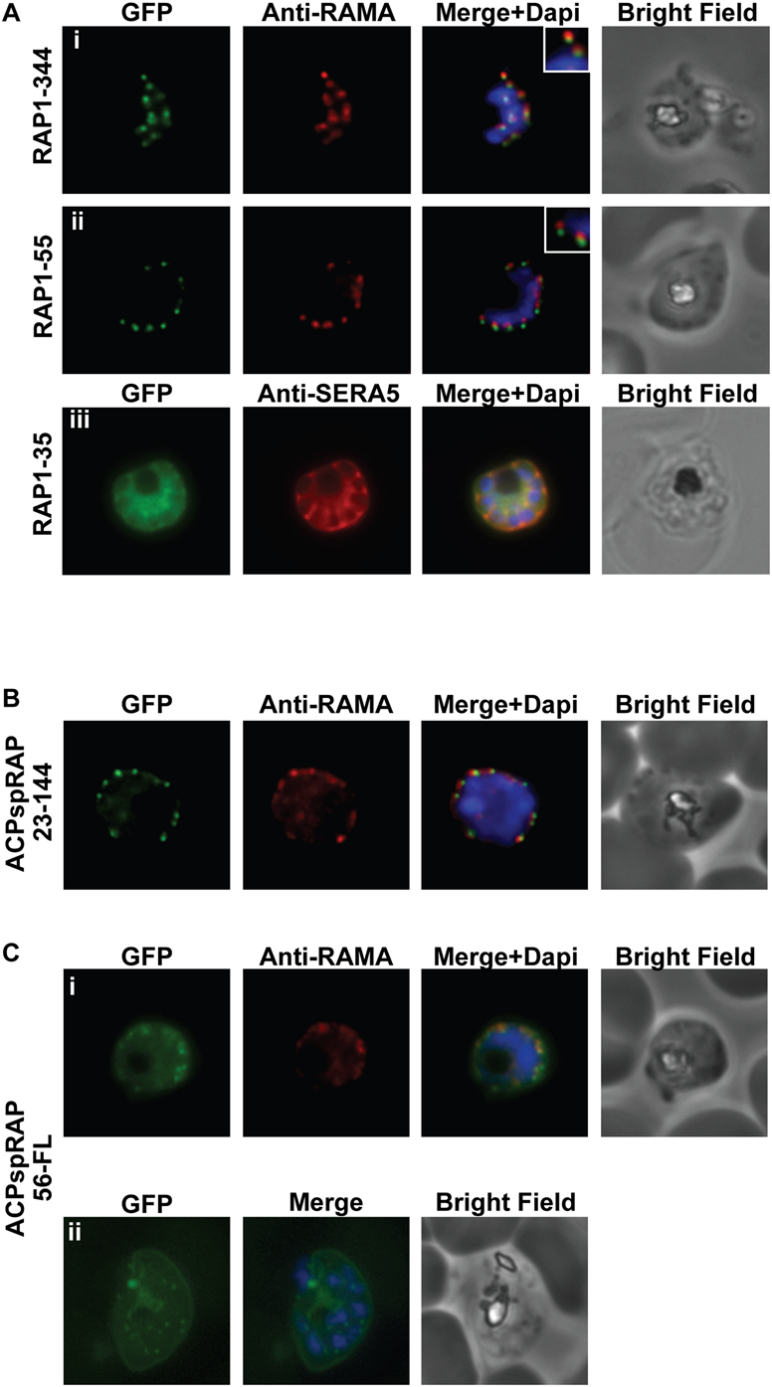
Amino acids 22-55 of RAP1 are sufficient and necessary for rhoptry targeting. (A) A series of RAP1 truncation-GFP fusions. The RAP1-344 and RAP1-55 constructs show a punctate fluorescence pattern characteristic of rhoptry localisation. GFP signal partially overlaps with RAMA staining, suggesting localisation in the rhoptry neck. For the RAP1-35 construct, GFP is trafficked to the PV as confirmed by co-localisation with SERA5. (B) Replacement of the predicted signal peptide in the RAP1-144 targeting construct with the ACP signal peptide (ACPspRAP23-144) has no effect on rhoptry localisation. (C) Fluorescence microscopy on parasites transfected with the ACPspRAP56-FL construct using fixed cells (i) or live cells (ii) demonstrates that deletion of the RAP1 rhoptry signal results in inefficient rhoptry targeting and the mistargeting of the bulk of the chimeric protein to the PV. PV staining was more obvious when the parasites were imaged without undergoing fixation.

Having confirmed the ability of RAP1-344 to target GFP to the rhoptries, we set out to define the minimal region sufficient for rhoptry targeting. To this end, we generated a series of N-terminal RAP1 truncation-GFP fusions (Figure 2A and S1). RAP1-244, RAP1-144, RAP1-65 and RAP1-55 were all able to direct trafficking of GFP to the rhoptries. In contrast, for the RAP1-35 construct, GFP fluorescence produced a ‘cluster of grapes’ pattern. Co-localisation with serine repeat antigen 5 (SERA5) (Figure 2Aiii), confirmed that RAP1-35-GFP was targeted to the parasitophorous vacuole (PV), the default destination for the secretory pathway [24],[25]. RAP1 possesses a typical N-terminal signal sequence that is presumably cleaved upon entry into the ER [31]. SignalP analysis of the RAP1 sequence predicts that this cleavage occurs between amino acids 21 and 22. Replacement of the RAP1 signal sequence with a signal sequence from the acyl carrier protein (ACP – normally targeted to the apicoplast) [24] had no effect on rhoptry localisation (Figure 2B). Our data strongly suggests that the signal sequence of RAP1 directs the protein into the secretory pathway. Information contained in amino acids 22-55 (hereafter referred to as the RAP1 rhoptry signal) is then sufficient to divert the protein to the rhoptries.

### Amino acids 22-55 of RAP1 are necessary for optimal rhoptry targeting

In *T. gondii*, proteins that are targeted to the rhoptries can contain multiple signals that are independently sufficient but not necessary for correct localisation [32],[33]. To determine whether this is the case for RAP1, we generated a construct that contains the ACP signal peptide fused to amino acids 56-782 of RAP1 (i.e. the entire RAP1 sequence lacking the signal peptide and the rhoptry signal) fused to GFP (Figure 2C). Although this construct was partially targeted to discrete foci that co-localised with RAMA, the bulk of the fluorescence was distributed in the PV. This data suggests that amino acids 22-55 of RAP1 are necessary for optimal targeting to the rhoptries.

### The N-terminus of RAP1 interacts with RAMA *in vitro*

**Figure 3:**
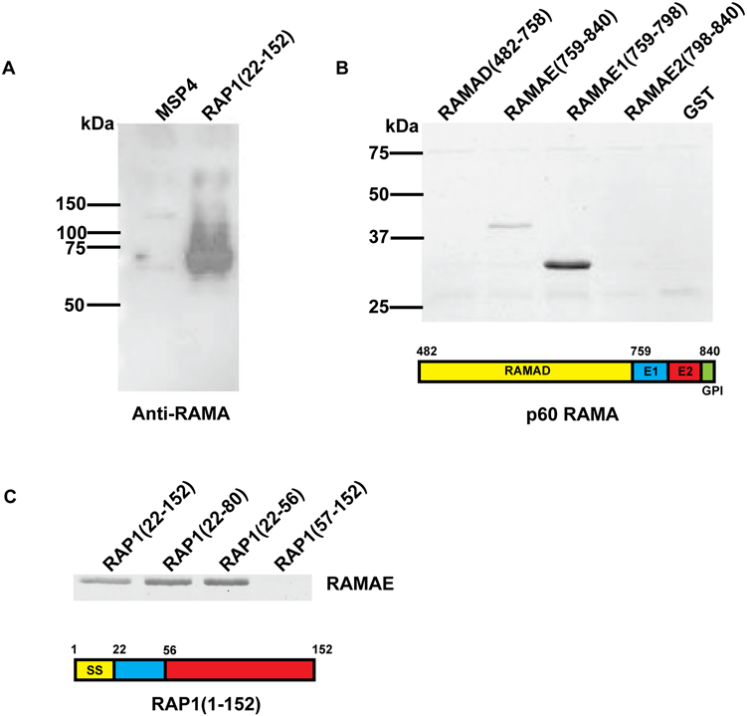
RAP1 recombinant proteins bind RAMA in pull-down assays. (A) RAP1(22-152) but not MSP4 (negative control) binds native RAMA p60 in parasite lysate. Binding of RAMA was detected by immunoblotting using anti-RAMA antibodies. (B) RAP1(22-152) binds to RAMAE and RAMAE1 GST-fusion proteins but not to the GST control. (C) Deletion of the RAP1 rhoptry signal from RAP1(22-152) abolishes binding of RAMAE. Schematics of RAMA and RAP1 fragments are shown; SS, signal sequence.

Having defined the RAP1 rhoptry signal, we were interested in the mechanism by which this region mediates targeting. Since RAMA is refractory to genetic deletion [34], we were unable to study the trafficking of RAP1 in RAMA deletion mutants. Furthermore, repeated attempts to overexpress full length RAMA, or RAMA lacking various domains (e.g. R1, R2 or R3 repeats) failed (results not shown), presumably due to toxic effects of overexpression of this protein. Instead, we decided to map the RAMA-RAP1 interaction *in vitro*. We reasoned that if RAMA acts as an escorter for the LMW complex, it should interact with the RAP1 rhoptry signal which is responsible for correct targeting of the complex. To test this hypothesis, we initially made a recombinant His_6_-tagged RAP1 protein representing amino acids 22-152 (RAP1(22-152)), which contains within it the RAP1 rhoptry signal, and used it in a pull-down assay (Figure 3). Our results indicate that RAP1(22-152) but not MSP4 (an irrelevant His_6_-tagged protein) bound RAMA in a schizont stage parasite extract (Figure 3A). To confirm these findings and more precisely map the RAP1 binding site within RAMA, we made RAMA-GST fusion proteins representing amino acids 482-758 (RAMAD), 759-840 (RAMAE), 759-798 (RAMAE1) and 799-840 (RAMAE2). We used these proteins together with RAP1(22-152) in pull-down assays (Figure 3B). RAMAE and RAMAE1 both bound to RAP1(22-152), whilst GST alone did not bind. Truncation of the C-terminus of RAP1(22-152) did not affect RAMAE binding, whereas deletion of the RAP1 rhoptry signal from RAP1(22-152) (construct RAP1(57-152)) abolished RAMAE binding (Figure 3C). Taken together these results demonstrate that the RAP1 rhoptry signal, involved in the targeting of the LMW complex to the rhoptries, acts as the binding site for the C-terminus of RAMA.

### Mutation of aromatic amino acids that affect the RAP1-RAMA interaction also affect RAP1 targeting

**Figure 4:**
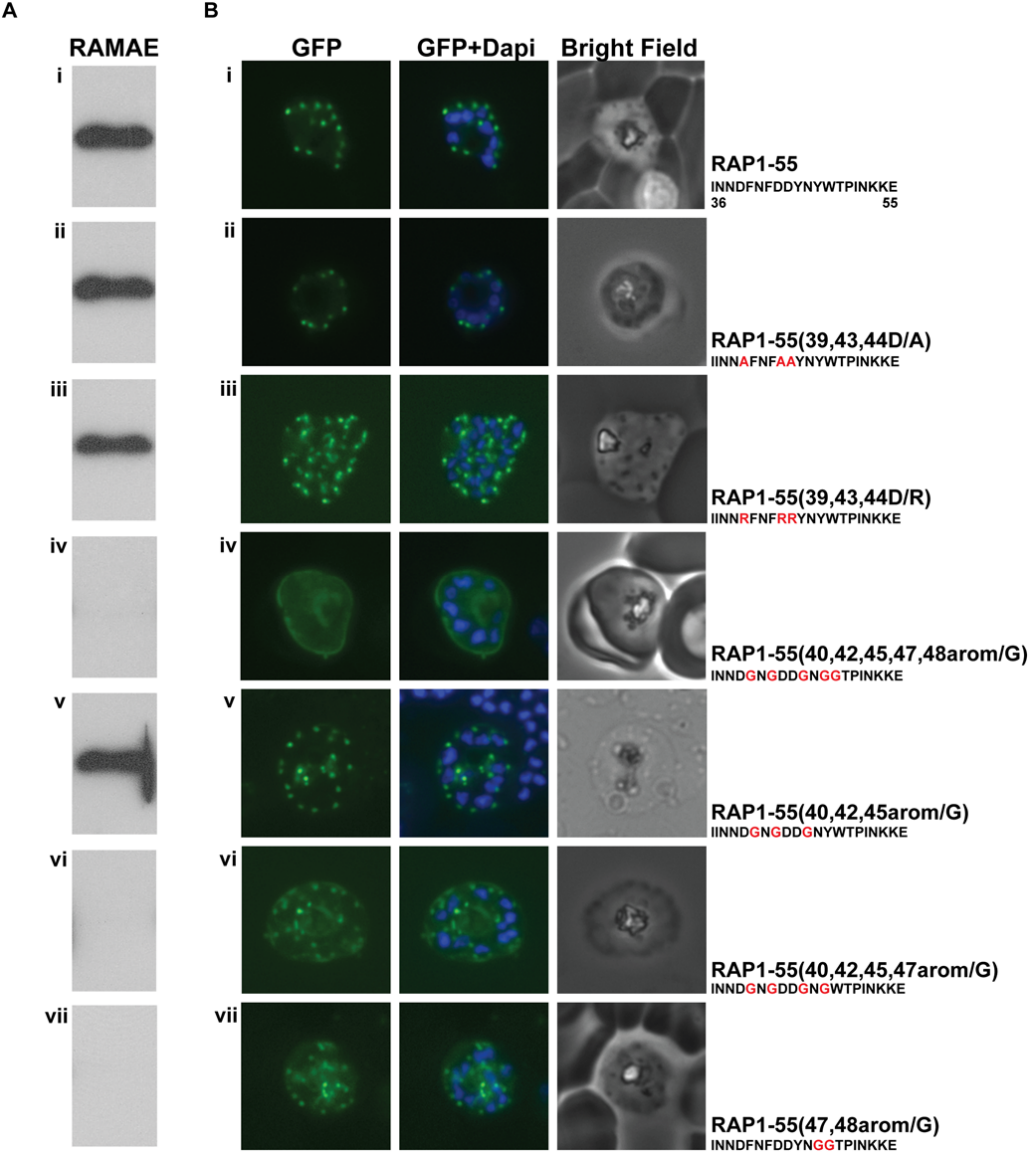
Mutation of aromatic amino acids that abolishes the RAMA-RAP1 interaction also interferes with RAP1 targeting. The same residues were mutated in the RAP1-55 targeting construct and in RAP1(22-152) recombinant protein, and the mutants were tested for their ability to bind RAMAE in a pull-down assay (A) or to target GFP to rhoptries (B). Binding of RAMAE was detected by immunoblotting using anti-GST antibodies. Mutated residues are shown in red. Mutation of all five aromatic residues in the RAP1 rhoptry signal completely abolished RAMAE binding and RAP1 targeting.

We had mapped the RAP1 rhoptry signal and the RAMA binding site to the N-terminus of RAP1. Due to low expression levels of RAP1-GFP chimeras we could not directly confirm RAMA binding by immunoprecipitation. Instead, we made a series of RAP1(22-152) mutant proteins and tested them in pull-down assays against RAMAE (Figure 4A). The same amino acids were also mutated in the RAP1-55 targeting construct so that their affect on RAP1 targeting *in vivo* could be examined (Figure 4B and S2) We focussed on residues 30–55 as these contain at least part of the information required for correct trafficking of RAP1. Amino acid alignment of RAP1 orthologues from different *Plasmodium* spp. failed to identify any potential conserved motifs within the RAP1 rhoptry signal (data not shown). Mutation of negatively charged residues (aspartate 39, 43 and 44) to either non-polar (alanine) or positively charged (arginine) residues failed to disrupt either rhoptry targeting or RAMA binding (Figure 4ii and 4iii). By contrast, mutation of aromatic residues (at positions 40, 42, 45, 47 and 48) to glycine abolished the RAMA-RAP1 interaction and resulted in mistargeting of GFP to the PV (Figure 4iv).

To analyse the individual importance of each of the aromatic residues we made mutants where only some of the aromatic residues were changed to glycines. Mutation of residues 40, 42, and 45 was insufficient to alter either RAMA binding or *in vivo* targeting (Figure 4Av and 4Bv). Simultaneous mutation of residues 40, 42, 45 and 47 or 47 and 48 abolished RAMA binding (Figure 4Avi and 4Avii). The same mutations in the RAP1-55 targeting constructs resulted in significant mistargeting of GFP to the PV, although some chimeric GFP could be observed in rhoptries (Figure 4Bvi and 4Bvii). This is likely a reflection of the sensitivity of the *in vitro* assay. *In vivo*, the reduced affinity of the interaction results in partial mistargeting, whereas *in vitro* the interaction falls below detectable levels. These results indicate that although residues 47 (tyrosine) and 48 (tryptophan) play a significant role in RAP1 targeting, it is the overall nature of the RAP1 rhoptry signal that is important.

### Proteolytic processing of the RAMA-RAP complex

**Figure 5:**
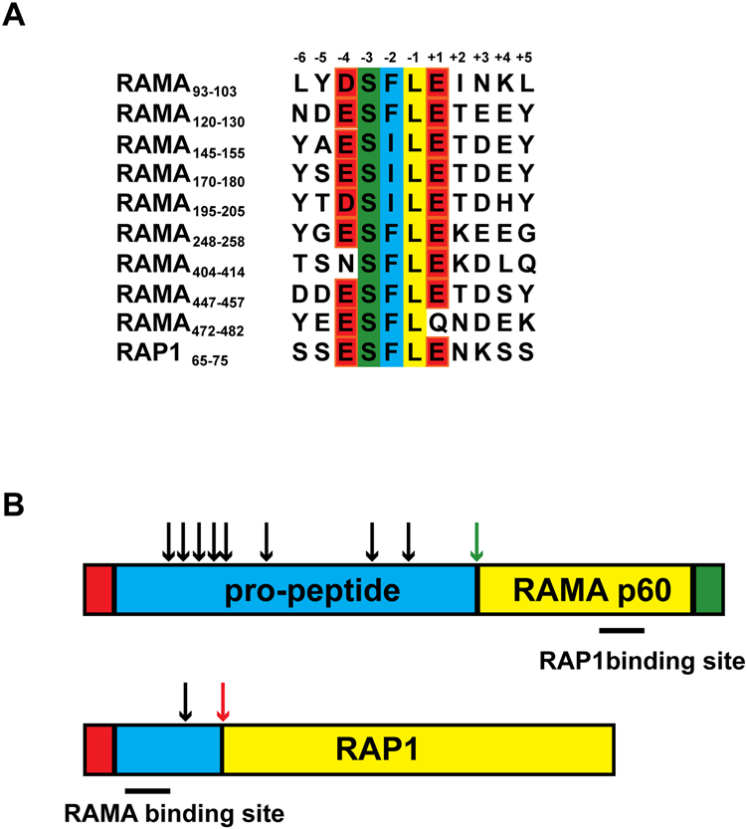
Putative protease cleavage sites for processing of RAMA and RAP1 within the rhoptries. (A) Amino acid alignment of putative protease cleavage sites within RAMA and RAP1. The alignment was generated using KALIGN. (B) Schematic of putative cleavage sites within RAMA and RAP1. Red shaded areas represent the signal peptide; blue shaded areas represent the pro-peptide, green shaded area represents the GPI anchor, and yellow shaded areas represent the mature protein. Putative cleavage sites are indicated with black arrows. The p60 RAMA cleavage site has been mapped by N-terminal sequencing (green arrow) [39]. Cleavage of the RAP1 pro-peptide is known to occur upstream of amino acid 124 (red arrow) [38]. RAP1 and RAMA binding sites are indicated.

The interaction between RAMA and RAP1 *in vivo* was initially demonstrated by FRET, a technique that measures photon transfer between two fluorophores that are in close proximity [15]. In our attempts to affinity purify the RAMA-RAP complex from schizont stage parasites, we found that only a small amount of RAMA co-precipitated with RAP1, and vice versa (results not shown). This data is consistent with previous studies [21], [35]–[37], and suggests that the RAMA-RAP interaction is transient. Both RAMA and RAP1 are synthesised as pre-proteins that are proteolytically processed within nascent rhoptries, presumably by a rhoptry-resident protease [15],[38]. We hypothesised that this processing may serve to dissociate the transient RAMA-RAP complex. The N-terminal pro-peptide of RAMA is unusually large and comprises more than 50% of the entire protein [15]. The N-terminus of the mature RAMA protein (RAMA p60) has recently been mapped using N-terminal sequencing (cleavage occurs between residues 477L and 478Q). Analysis of RAMA orthologues from different *Plasmodium* spp. indicates that the protease responsible for this cleavage recognises the sequence (D/E)SFL(Q/E) [39]. We examined the primary structure of RAMA and found that this sequence and/or closely related sequences are repeated eight times within the pro-peptide region but are not present within RAMA p60 (Figure 5). A putative cleavage site was also identified at amino acids 67-71 (ESFLE) of RAP1. Cleavage of the RAP1 pro-peptide has been mapped upstream of A124 and involves the removal of approximately 40 amino acids (in addition to the signal peptide) [38]. We attempted N-terminal sequencing of immunoaffinity-purified RAP1, but did not obtain any data presumably due to N-terminal blockage of the protein. We also performed a trypsin digestion and liquid chromatography-mass spectrometry (LC-MS) analysis. In two independent analyses, we obtained >60% coverage of RAP1 downstream of the putative cleavage site, but did not detect any peptides upstream of the cleavage site. The most N-terminal peptide detected corresponded to amino acids 74-91 of RAP1 (results not shown). The peptide corresponding to amino acids 71-73 (which would be present if cleavage occurs between 70L and 71E) is too small to be detected. This data, in combination with previously published data, strongly suggests that both RAMA and RAP1 are processed by the same rhoptry-resident protease.

### Amino acids 344-444 of RAP1 contain a PV transfer motif

**Figure 6:**
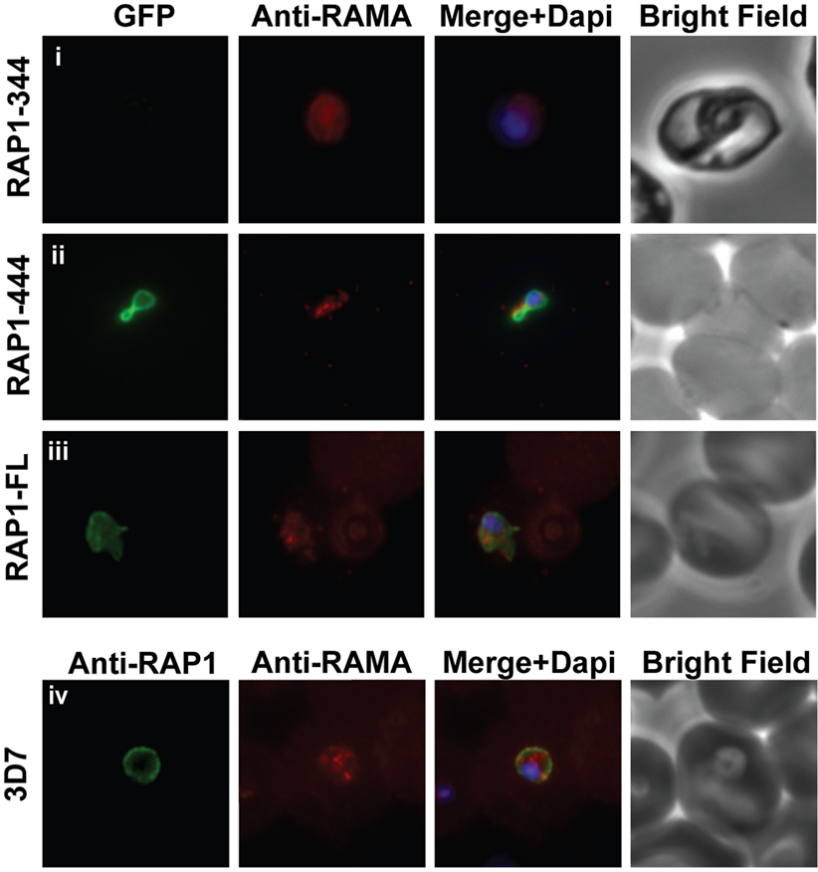
Amino acids 344-444 of RAP1 are required for the transfer of RAP1 to the PV during invasion. For the RAP1-444 (ii) and RAP1-FL (iii) constructs, the GFP chimeras are transferred to the PV of newly formed ring stage parasites and mimic the localisation of native RAP1 in wild-type 3D7 parasites (iv). In contrast, the RAP1-344 (i) construct is not transferred to the PV during invasion.

Many, though not all, rhoptry proteins are secreted during merozoite invasion and are transferred to the PV of nascent ring stage parasites where they presumably play a role in the establishment of the PV membrane [40]. Earlier studies using D10ΔRAP1, have demonstrated that full length RAP1 is transferred to the PV during invasion, whereas truncated RAP1 is not [22]. Given our finding that the C-terminus of RAP1 contains a rhoptry bulb retention motif (see above), it is possible that RAP1 secretion is dependent on correct sub-organellar localisation. To more precisely map the signals within RAP1 required for rhoptry bulb retention and PV transfer, we generated a further series of RAP1 truncation-GFP fusions that included regions of the C-terminus of the protein. These parasites were examined by fluorescence microscopy both at schizont stage (to establish rhoptry bulb or rhoptry neck localisation) and at ring stage (to ascertain transfer to the PV). As expected, RAP1-344GFP, which is localised in the rhoptry neck (Figure 1Bii), was not transferred to the PV during invasion (Figure 6i). In contrast, for the full-length RAP1, RAP1-644 and RAP1-544 constructs, chimeric GFP was localised in the rhoptry bulb at schizont stage (Figure 1Aii and S3), and could be observed as a rim of fluorescence around newly formed ring-stage parasites indicating transfer to the PV (Figure 6iii and S3). The RAP1-444GFP chimera appeared to be only partially localised in the rhoptry bulb (Figure S3), but was nonetheless transferred to the PV during invasion (Figure 6ii). These results indicate that amino acids 344-444 of RAP1 are required for transfer of the protein to the PV. Our attempts to confirm sub-organellar localisation of the RAP1-GFP chimeras using immunoelectron microscopy were unsuccessful due to the relatively low level of expression of episomal constructs. However, our confocal microscopy results provide preliminary evidence that amino acids 344-544 of RAP1 are required for correct sub-organellar localisation of the protein within the rhoptry.

## Discussion

Apical organelles of apicomplexan parasites play a key role in invasion of target cells and the subversion of host cell function. Rhoptries of *P. falciparum* merozoites contain a complex proteome including components that have been identified as potential vaccine candidates (reviewed in [6]). However, little is known about mechanisms of rhoptry biogenesis and discharge.

In the present study, we examined the trafficking of the rhoptry protein RAP1. RAP1, together with RAP2 or RAP3, form the heterodimeric LMW complex which is localised in the rhoptry bulb of schizonts [21],[23]. During invasion, the LMW complex is secreted from the rhoptries and transferred to the PV of the nascent ring-stage parasite [35]. Truncation of the C-terminus of RAP1 results in disruption of its interaction with RAP2/RAP3 and causes RAP2 (and probably RAP3) to be retained in the ER [22]. In contrast, truncated RAP1 is still targeted to rhoptries, but is not transferred to the PV during invasion [22].

Our results confirm and expand on these earlier observations. Using expression of GFP chimeras we were able to show that information present between amino acids 23 and 55 of RAP1 is necessary and sufficient for optimal targeting to the rhoptries. We compared the RAP1 rhoptry signal to protein regions that have been implicated in rhoptry targeting in *Toxoplasma*, as well as other *Plasmodium* rhoptry proteins but were unable to identify a conserved motif. This suggests that the RAP1 rhoptry signal is specific for the LMW complex and it may be that unlike proteins targeted to the apicoplast [41] or exported into the host RBC [42],[43], many proteins destined for the rhoptries do not possess a common targeting signal. We have provided evidence that RAMA, a protein synthesised in the late ring stage and GPI-anchored in the Golgi lumen, acts as an escorter for the LMW complex via a direct association with the N-terminus of RAP1. Bulky aromatic amino acid clusters are known to be important for protein-protein interactions. In the case of the RAMA-RAP1 interaction, it appears that the overall organisation of aromatic residues within the RAP1 rhoptry signal is important for correct binding. However, in the absence of structural information, we cannot determine whether any or all of these residues directly contact RAMA, or whether disruption of RAMA binding and mistargeting in our mutants occurs as a result of conformational perturbation caused by glycine substitution.

RAP1 appears to possess a distinct signal for localisation within the rhoptry bulb and subsequent transfer to the PV during invasion. These findings are consistent with an earlier study in *Toxoplasma* which demonstrated that the pro-domain of the rhoptry protein ROP1 directs trafficking of a reporter to the rhoptry neck, whereas full-length ROP1 is preferentially enriched in the bulb [44]. The mechanism by which proteins can be partitioned within a single membrane bound organelle is not understood. Our data argues for the presence of a bulb-retention motif within the C-terminus of RAP1 which may allow interaction with other rhoptry bulb proteins (e.g. RAP2 and −3). It is worth noting that RAP1 is a major constituent of detergent-resistant microdomains (DRMs) in schizont stage parasites [37]. RAP1 has no obvious lipid anchor and is likely recruited into DRMs via association with some other protein.

Whether or not localisation of RAP1 in the rhoptry bulb is necessary for transfer of the protein to the PV *per se*, is not clear. One possibility is that rhoptry neck proteins are secreted before rhoptry bulb proteins and are deposited onto the surface of the target RBC. In contrast, rhoptry bulb proteins are trapped within the PV because their secretion occurs after the formation of the tight junction between the parasite membrane and the RBC membrane. The alternative explanation is that amino acids 344-444 of RAP1 contain a specific protein-protein interaction motif (e.g. necessary for interaction with RAP2 and −3) which is required for transfer to the PV. Detailed mapping of other sub-organellar localisation signals and PV transfer signals will help to differentiate between these two alternatives.

**Figure 7:**
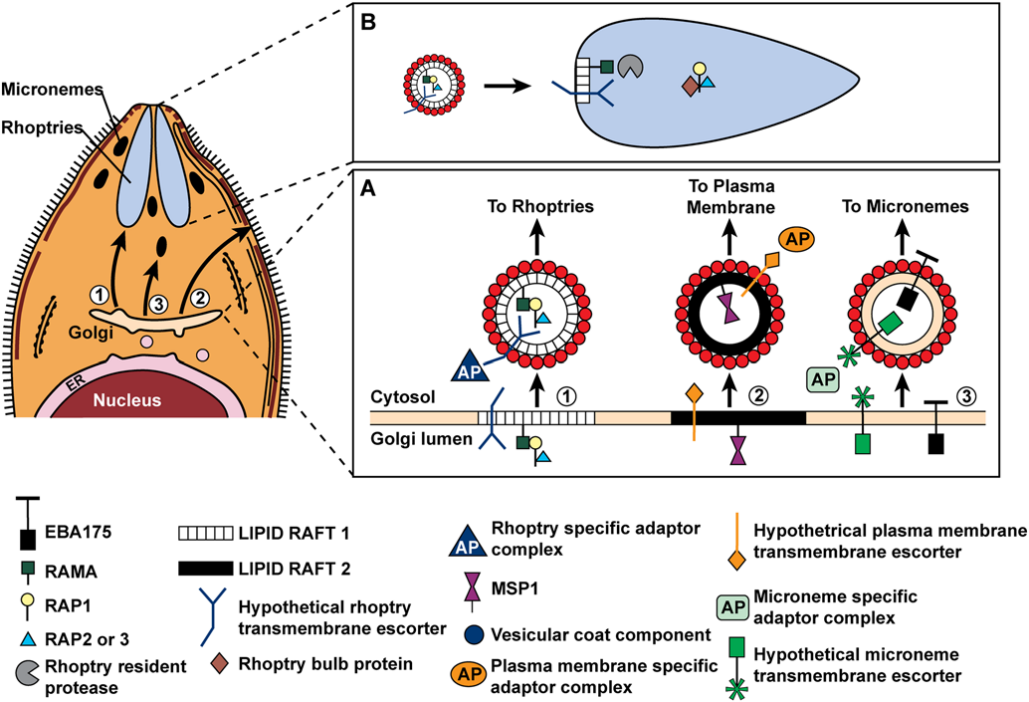
A proposed model for targeting of proteins to the rhoptries. (A) Proteins destined for the plasma membrane or the apical organelles are co-translationally inserted into the ER and are trafficked to the Golgi. Within the Golgi, proteins destined for the rhoptries (1), the plasma membrane (2), or the micronemes (3) aggregate into distinct sub-domains. Rhoptries and plasma membrane proteins are probably clustered in lipid rafts whereas microneme proteins are excluded from lipid rafts. Specific escorters that are exposed on the cytoplasmic face of the Golgi recruit adaptor and vesicle coat proteins. The sub-domains bud off as individual vesicles and are directed to their respective destinations by specific interactions with the vesicular trafficking machinery. (B) Fusion of the vesicle with the rhoptry delivers the proteins into the rhoptry lumen. Proteolytic processing of RAMA and RAP1 by a rhoptry resident protease releases the proteins from the transient trafficking complex. The LMW complex is retained in the rhoptry bulb via its bulb-retention motif.

Based on the data presented above, we propose a model whereby RAMA binds RAP1 in the Golgi lumen and recruits RAP1, −2 and −3 into a complex (Figure 7). GPI-anchored proteins have a tendency to cluster in lipid rafts, and thus the complex is presumably anchored within a lipid raft at the Golgi exit face [45],[46]. Other proteins (e.g. the RhopH proteins which also interact with RAMA) may be recruited into the raft as well, thus generating rhoptry destined aggregates [15].

DRM clustering associated with protein oligomerisation has been shown to be essential for polarised trafficking of GPI-anchored proteins to the apical membrane in epithelial cells (reviewed in [47],[48]). Several of the known *P. falciparum* rhoptry proteins, including RAMA and RAP1, are associated with DRMs and it is tempting to speculate that this mechanism is involved in differential sorting of proteins within the Golgi. Interestingly, none of the known micronemal proteins have been found associated with DRMs, whereas several merozoite surface proteins do associate with DRMs [45]. This suggests the presence of distinct regions of membrane at the Golgi exit face which are defined by their protein and/or lipid composition that bud off as individual vesicles. Each vesicle then presumably interacts with specific components of the cellular trafficking machinery, possibly via a transmembrane escorter. In *Toxoplasma*, the cytoplasmic adaptor complex AP-1 has been implicated in rhoptry protein trafficking. A study by Hoppe and colleagues demonstrated that AP-1 binds *in vitro* to a region of the *Toxoplasma* rhoptry protein ROP2 that is sufficient to mediate rhoptry targeting *in vivo* [18]. The biological relevance of this finding has recently come into question as ROP2 appears to lack a transmembrane domain that is necessary in order for the ROP2 targeting region to be exposed at the Golgi exit face and available for binding to AP-1 [49]. Nonetheless, components of vesicular trafficking machinery, including AP-1, have been identified in the *P. falciparum* genome but their precise roles remain to be determined (reviewed in [40]).

Upon arrival at the rhoptry, the RAMA-LMW complex is dissociated by proteolytic cleavage [15],[38]. The presence of putative cleavage sites in the N-terminus of RAP1 and RAMA suggests that a single rhoptry-resident protease is responsible for their processing [39]. Cleavage of the N-terminus of RAP1 releases the LMW complex from RAMA. This may allow the LMW complex to interact with other proteins in the rhoptry bulb, potentially via the bulb-retention domain of RAP1 identified in this study [37]. In turn, degradation of the N-terminus of RAMA may release it from the hypothetical transmembrane escorter.

Proteins destined for the apicoplast or mitochondrion each possess an appropriate signal that allows their post-translational translocation into a pre-formed organelle [41], [50]–[53]. In contrast, many proteins destined for the apical secretory organelles appear to be targeted by a clustering mechanism. In *Toxoplasma*, the soluble microneme proteins MIC1, MIC3 and MIC4 are targeted via an interaction with transmembrane escorter proteins [54],[55]. In *Plasmodium*, microneme proteins of the EBL family are targeted courtesy of a conserved luminal domain presumably via interaction with a transmembrane escorter [56],[57]. In the current study, we present evidence that proteins can be similarly targeted to rhoptries via the formation of transient complexes that are packaged into transport vesicles and dissociated by proteolytic processing upon arrival at their destination. Given that most *Plasmodium* rhoptry proteins are not type 1 membrane proteins and therefore lack a cytoplasmic tail, it is likely that targeting to rhoptries via this mechanism is the rule rather than the exception.

## Materials and Methods

### Parasite cultures

*P. falciparum* asexual stage parasites were maintained in human erythrocytes (blood group O+) at a hematocrit of 4% with 10% Albumax (Invitrogen) [58]. *P. falciparum* 3D7 parasites were originally obtained from David Walliker at Edinburgh University. Cultures were synchronised as previously described [59].

### Cloning

All oligonucleotide primers used in this study are listed in Table S1. GFP fusion proteins for localization studies were encoded in transfection constructs under the regulation of the tetracycline-inducible expression system [26]. Regions of RAP1 were PCR amplified from *P. falciparum* 3D7 genomic DNA. For mutagenesis experiments, mutations were introduced into primers during synthesis. PCR products were digested with *Pst*I and *Mlu*I and cloned in frame upstream of GFP. RAMAE1 and RAMAE2 recombinant fragments were PCR amplified from *P. falciparum* cDNA and cloned as previously described [15] into the GST-fusion vector pGEX-4T-1 (GE Healthcare). RAP1 recombinant proteins were PCR amplified from *P. falciparum* 3D7 genomic DNA. PCR products were digested with *Nco*I and *Xho*I and cloned into the His_6_-fusion vector pET28b in frame upstream of the His_6_ tag. Constructs were sequenced and confirmed to be free of unintended mutations.

### Recombinant protein expression

His_6_-tagged RAP1 recombinant proteins were expressed in *E. coli* BL21 (DE3) (Novagen) and purified using TALON Metal Affinity Resin (Clontech) in accordance with manufacturer's instructions. RAMA-GST fusion proteins were expressed in *E. coli* BL21 (DE3) and purified using glutathione resin (Sigma) as previously described [15]. Protein expression was analysed using SDS-PAGE and immunoblotting with anti-His_6_ or anti-GST antibodies. Protein concentration was determined using the Bradford Assay (Bio-Rad).

### Pull-down assay

Purified recombinant proteins were buffer exchanged into pull-down buffer (50 mM Na_2_HPO_4_, 75 mM NaCl, 0.1% TrionX-100, 5 mM imidazole, pH 7.4). *P. falciparum* 3D7 parasites were extracted from parasitised RBCs by lysis with 0.15% (w/v) saponin in phosphate buffered saline and solubilised in RIPA buffer (50 mM TrisCl (pH 8.8), 150 mM NaCl, 1% NP40, 0.5% sodium deoxycholate, 0.1% SDS) containing Complete Mini protease inhibitor cocktail (Roche). After lysis on ice for 5 min the insoluble material was spun down and the supernatant collected. The supernatant was diluted 1 part in 10 in pull down buffer prior to use. For the pull-down assay, 100 µg of the various His_6_-tagged RAP1 recombinant proteins were used as bait. These proteins were immobilised on TALON Metal Affinity Resin (Clontech) and incubated with either GST-fusion proteins or parasite lysate O/N at 4°C. The resin was washed with pull-down buffer and specifically bound proteins were eluted using imidazole (20 mM Na_2_HPO_4_, 0.5 M NaCl, 400 mM imidazole, pH 7.4). Eluted proteins were analysed on Coomassie stained SDS-PAGE gels and by immunoblotting with anti-RAMA [15] or anti-GST antibodies.

### Parasite Transfection

*P. falciparum* 3D7 parasites were transfected as described previously [52] with 100 µg of purified plasmid DNA (Qiagen). Positive selection for transfectants was achieved using 10 nM WR99210 and 0.5 µg/ml Anhydrotetracycline to prevent transgene expression.

### Fluorescence Imaging

Anhydrotetracycline was removed from parasite cultures 72 h prior to live imaging (in the presence of 10 nM WR99210) to allow expression of the GFP fusion. Prior to microscopy, parasites were incubated in culture medium containing 100 ng/ml 4′,6-diamidino-2-phenylindole (DAPI; Roche Molecular Biochemicals). Fluorescence images of schizont stage parasites were captured using a Carl Zeiss Axioskop microscope with a PCO Sensicam and Axiovision 2 software. For immunofluorescence assays, schizont stage parasites were fixed using 4% paraformaldehyde (ProSciTech) and 0.0075% glutaraldehyde (ProSciTech) as previously described [41]. After blocking in 3% bovine serum albumin (Sigma) the cells were incubated for 1 hour with rabbit anti-RAMA [15], mouse anti-AMA1 [60], mouse anti-RAP1 or rabbit anti-PfRON4 (Richard and Cowman, manuscript in preparation) antibodies. Bound antibodies were then visualised with Alexa Fluor-594 anti-rabbit IgG or anti-mouse IgG (Molecular Probes) diluted 1∶1000. Parasites were mounted in Vectashield (Vecta Laboratories) containing DAPI.

### Immunoelectron Microscopy

Parasites for electron microscopy immunolabeling were fixed and prepared as described previously (Healer et al., 2002). The primary antibodies used were mouse monoclonal anti-PfRAP-1 (1/500), rabbit anti-PfRON-4 (1/100). Samples were washed, then incubated with secondary antibodies conjugated to either 10 nm or 15 nm colloidal gold (BB International). Samples were then post-stained with 2% aqueous uranyl-acetate then 5% triple lead and observed at 120 kV on a Philips CM120 BioTWIN Transmission Electron Microscope.

## Supporting Information

Table S1 Oligonucleotide primers used in this study
(0.05 MB DOC)

Figure S1 A series of RAP1 truncation-GFP fusions co-localised with the rhoptry bulb marker RAMA
(1.52 MB TIF)

Figure S2 Co-localisation of RAP1 truncation-GFP fusions with the rhoptry bulb marker RAMA. Mutated residues are shown in parentheses.
(2.24 MB TIF)

Figure S3 For the RAP1-544 and RAP1-644 constructs, GFP chimeras are localised in the rhoptry bulb (A) and are transferred to the PV of nascent ring stage parasites (B); and, for the RAP1-444 construct, the GFP chimera is only partially localised in the rhoptry bulb.
(2.12 MB TIF)
