# Supplementary material for: Identification of Rhoptry Trafficking Determinants and Evidence for a Novel Sorting Mechanism in the Malaria Parasite Plasmodium falciparum
Source: PLoS Pathog. 2009 Mar 6;5(3):e1000328. doi: 10.1371/journal.ppat.1000328 (PMC2648313; doi:10.1371/journal.ppat.1000328)
Supplement: Table S1 — Oligonucleotide primers used in this study (0.05 MB DOC) [file ppat.1000328.s001.doc]

| **Primer sequence** | **Description** | **Restriction site** |
| --- | --- | --- |
| 5'tcgccatgggtataaatgtaaacgg3' | RAP1(22-152) fwd | *Nco*I |
| 5'tggctcgagtgactttgtactagatgg3' | RAP1(22-152) rev | *Xho*I |
| 5'attccatggaagataaattttcaagtg3' | RAP1(57-152) fwd | *Nco*I |
| 5'tttctcgagataggaatttaaaaattcc3' | RAP1(22-56) rev | *Xho*I |
| 5'taactcgagatttccatcatcaacactag3' | RAP1(22-80) rev | *Xho*I |
| 5'tcgccatgggtataaatgtaaacggagataataattatgggaaaacaataatcaataatgctttcaattttgctgcttac3' | RAP1(22-152) (39,43,44 D/A) fwd | *Nco*I |
| 5'tcgccatgggtataaatgtaaacggagataataattatgggaaaacaataatcaataatcgtttcaattttcgtcgttac3' | RAP1(22-152)(39,43,44 D/R) fwd | *Nco*I |
| 5'tcgccatgggtataaatgtaaacggagataataattatgggaaaacaataatcaataatgatggcaattttg3' | RAP1(22-152)(40F/G) fwd | *Nco*I |
| 5'tcgccatgggtataaatgtaaacggagataataattatgggaaaacaataatcaataatgatggcaatggtgatgatggcaatggtgggac3' | RAP1(22-152)(40,42,45,47,48 arom/G) fwd | *Nco*I |
| 5'tcgccatgggtataaatgtaaacggagataataattatgggaaaacaataatcaataatgatggcaatggtgatgatggcaattattgg3' | RAP1(22-152)(40,42,45 arom/G) fwd | *Nco*I |
| 5'tcgccatgggtataaatgtaaacggagataataattatgggaaaacaataatcaataatgatggcaatggtgatgatggcaatggttggac3' | RAP1(22-152)(40,42,45,47 arom/G) fwd | *Nco*I |
| 5'tcgccatgggtataaatgtaaacggagataataattatgggaaaacaataatcaataatgatttcaattttgatgattacaatggtgggac3' | RAP1(22-152)(47, 48 aromatic/G) fwd | *Nco*I |
| 5'ctgcagtatatataatgagtttctatttgggta3' | RAP1 fwd | *Pst*I |
| 5'ctgcagtatatataatgaagatcttattactttgtataatttttctatattatgttaacgctttccatgtactcttccgtaatgtcg3' | ACPspRap23-144 fwd | *Pst*I |
| 5'ctgcagtatatataatgaagatcttattactttgtataatttttctatattatgttaacgcttttttaaattcctatgaagataaattttcaagtg3' | ACPspRap56-FL fwd | *Pst*I |
| 5'acgcgtgtgtcttggtactaattttaag3' | RAP1-644 rev | *Mlu*I |
| 5'acgcgttaattttcttgctttatatg3' | RAP1-544 rev | *Mlu*I |
| 5'acgcgttccatcagctaaagttaaataatc3' | RAP1-444 rev | *Mlu*I |
| 5'acgcgtatattcttgataggttacacc3' | RAP1-344 rev | *Mlu*I |
| 5'acgcgtaaattcttctttttgtgcaac3' | RAP1-244 rev | *Mlu*I |
| 5'acgcgtagatgattttaaaccagatg3' | RAP1-144 rev | *Mlu*I |
| 5'acgcgttgaaaatttatcttcatagg3' | RAP1-65 rev | *Mlu*I |
| 5'acgcgtttcctttttatttattggtgtcc3' | RAP1-55 rev | *Mlu*I |
| 5'acgcgtttcctttttatttattggtgtccaaccattgtaatcatcaaaattga5'aatcattattgattattgttttccc3' | RAP1-55(47Y/G) | *Mlu*I |
| 5'acgcgtttcctttttatttattggtgtccaataattaccatcatctccattaccatcattattgattattgttttccc3' | RAP1-55(40,42,45arom/G) | *Mlu*I |
| 5'acgcgtttcctttttatttattggtgtccatccattaccatcatctccattaccatcattattgattattgttttccc3' | RAP1-55(40,42,45,47arom/G) | *Mlu*I |
| 5'acgcgtttcctttttatttattggtgttcctccattaccatcatctccattaccatcattattgattattgttttccc3' | RAP1-55(40,42,45,47,48arom/G) | *Mlu*I |
| 5'acgcgtttcctttttatttattggtgttccaccattgtaatcatcaaaattgaaatcattattgattattgttttccc3' | RAP1-55(47,48arom/G) | *Mlu*I |
| 5'acgcgtttcctttttatttattggtgtccaataattgtaagcagcaaaattgaaagcattattgattattgttttccc3' | RAP1-55(39,43,44D/A) | *Mlu*I |
| 5'acgcgtttcctttttatttattggtgtccaataattgtaacgacgaaaattgaaacgattattgattattgttttccc3' | RAP1-55(39,43,44D/R) | *Mlu*I |
