# Supplementary figures and images for: Identification of Rhoptry Trafficking Determinants and Evidence for a Novel Sorting Mechanism in the Malaria Parasite Plasmodium falciparum
Source: PLoS Pathog. 2009 Mar 6;5(3):e1000328. doi: 10.1371/journal.ppat.1000328 (PMC2648313; doi:10.1371/journal.ppat.1000328)

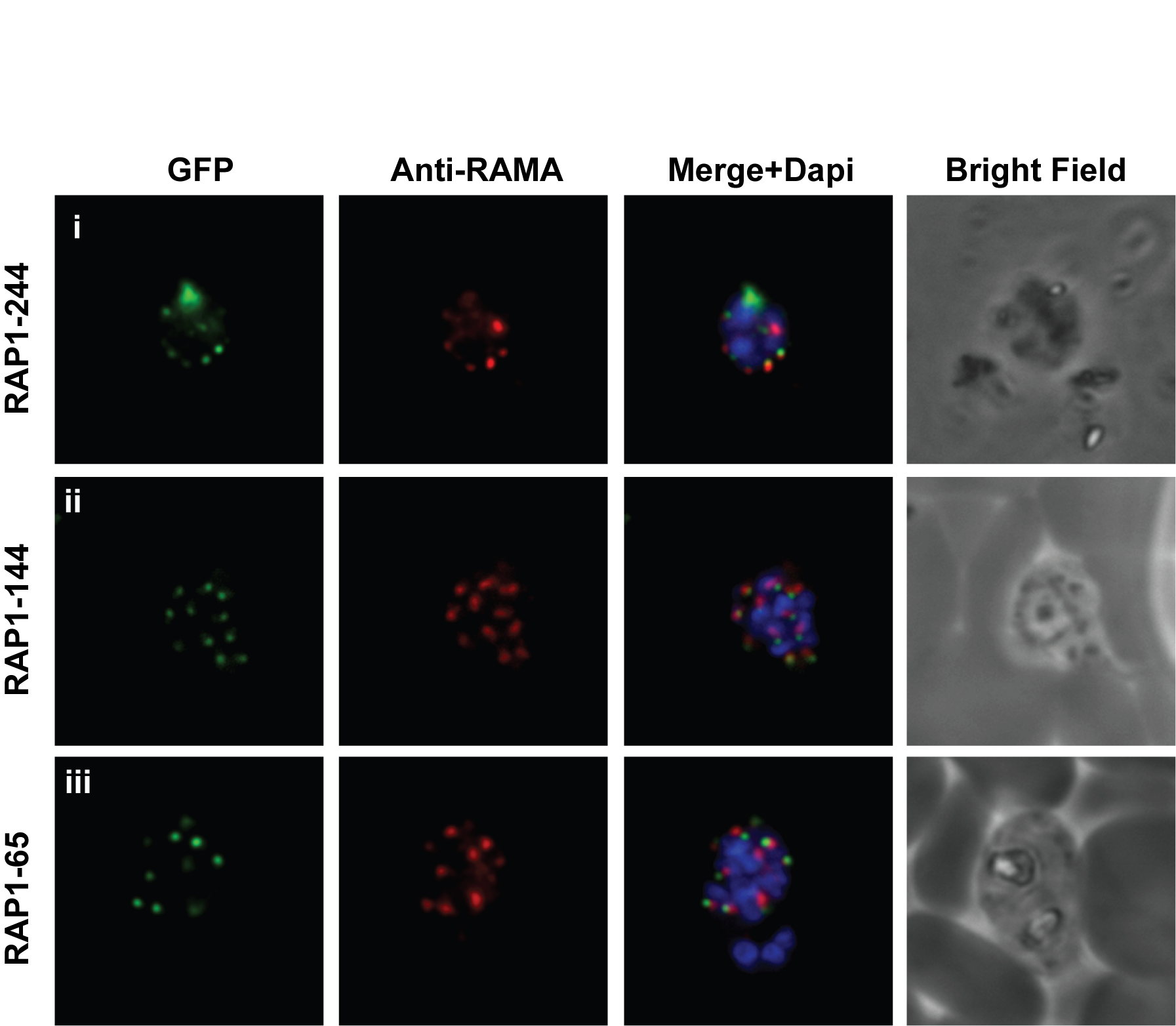

Supplement: Figure S1 — A series of RAP1 truncation-GFP fusions co-localised with the rhoptry bulb marker RAMA (1.52 MB TIF) [file ppat.1000328.s002.tif]

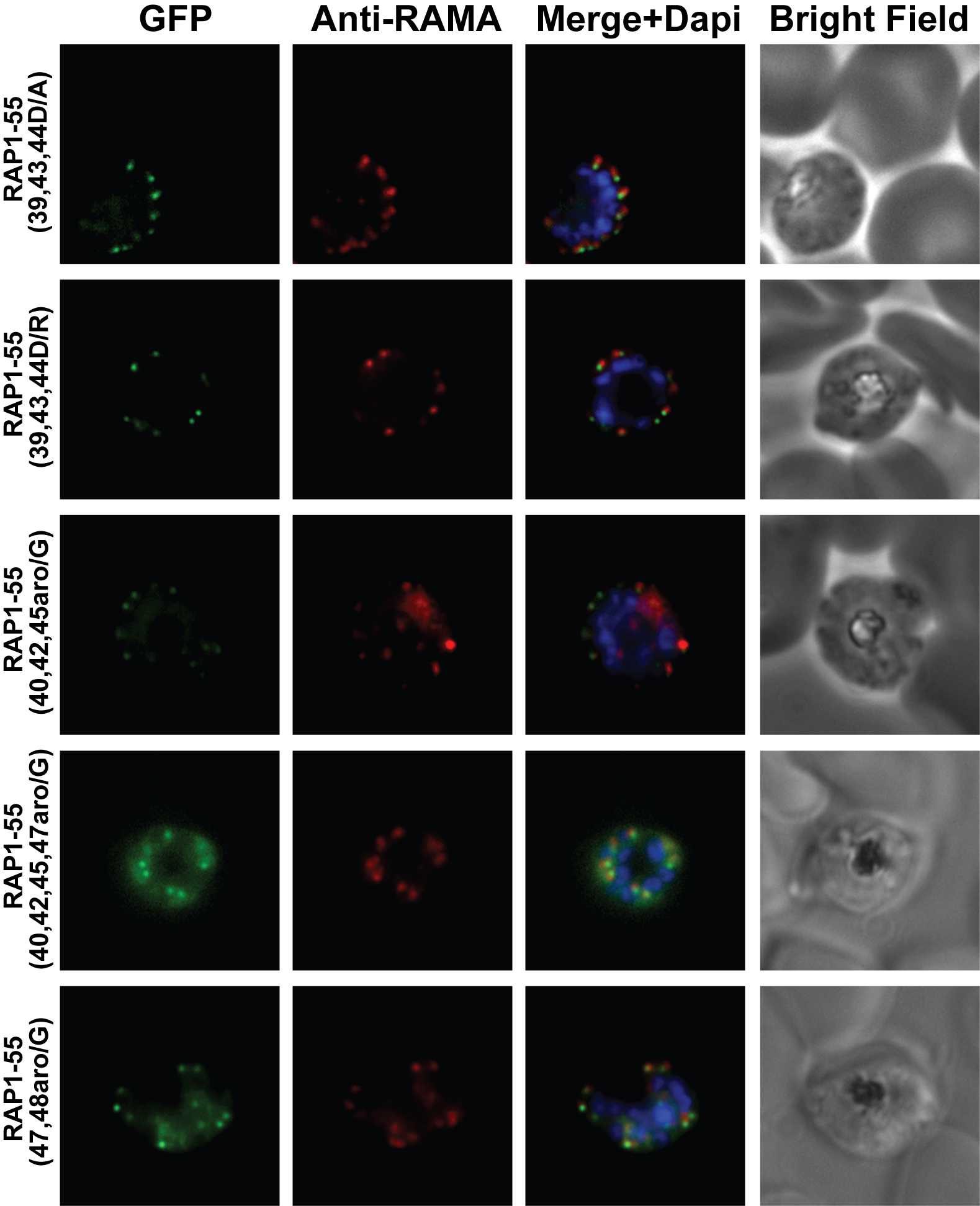

Supplement: Figure S2 — Co-localisation of RAP1 truncation-GFP fusions with the rhoptry bulb marker RAMA. Mutated residues are shown in parentheses. (2.24 MB TIF) [file ppat.1000328.s003.tif]

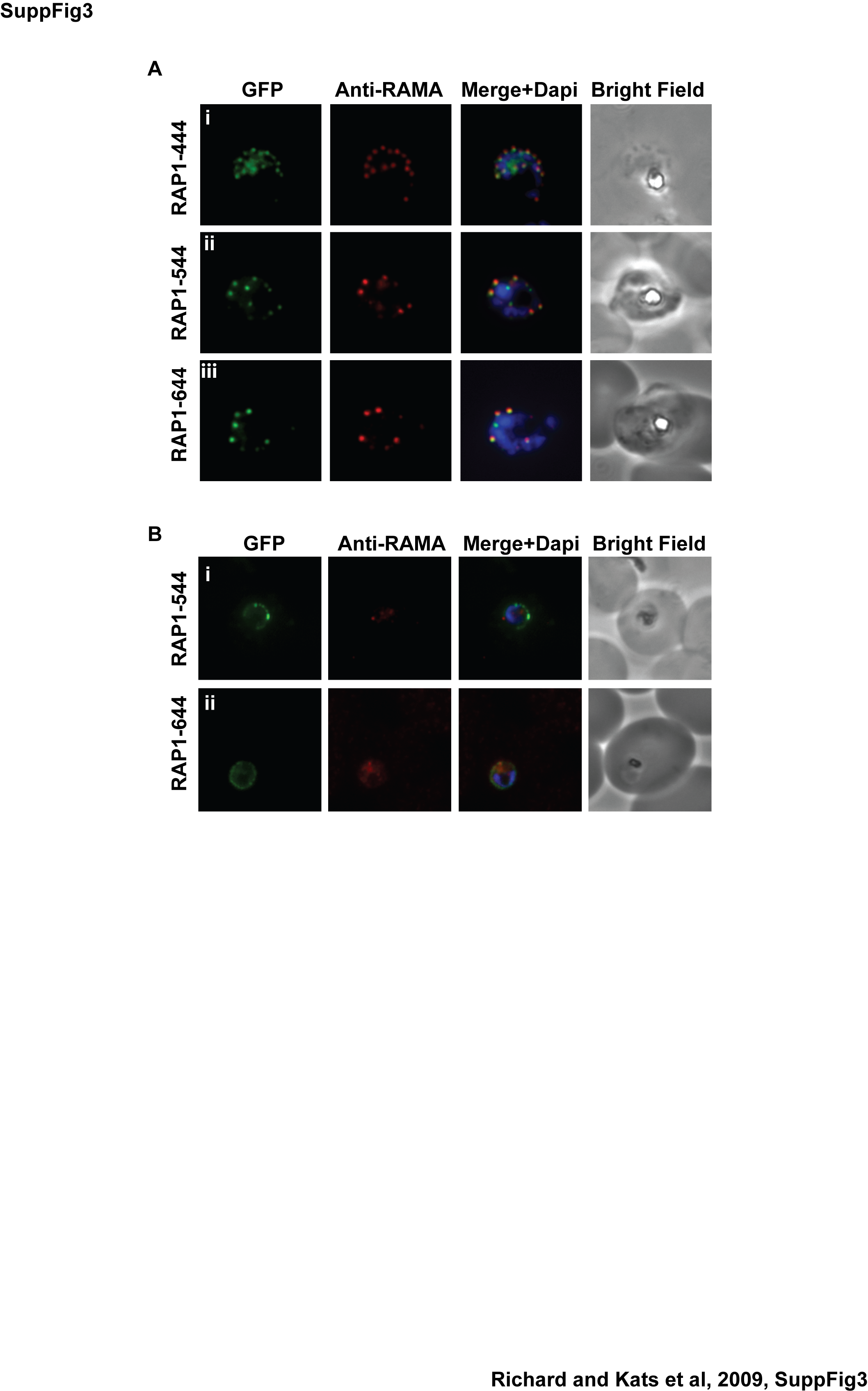

Supplement: Figure S3 — For the RAP1-544 and RAP1-644 constructs, GFP chimeras are localised in the rhoptry bulb (A) and are transferred to the PV of nascent ring stage parasites (B); and, for the RAP1-444 construct, the GFP chimera is only partially localised in the rhoptry bulb. (2.12 MB TIF) [file ppat.1000328.s004.tif]
